# High resolution tumor targeting in living mice by means of multispectral optoacoustic tomography

**DOI:** 10.1186/2191-219X-2-14

**Published:** 2012-04-01

**Authors:** Andreas Buehler, Eva Herzog, Angelica Ale, Bradley D Smith, Vasilis Ntziachristos, Daniel Razansky

**Affiliations:** 1 Institute for Biological and Medical Imaging, Technische Universität München und Helmholtz Zentrum München, Ingoldstädter Landstraße 1, Neuherberg 85764, Germany; 2 Notre Dame Integrated Imaging Facility, University of Notre Dame, Notre Dame, IN 46556-5670, USA

**Keywords:** Optoacoustic imaging, Tumor targeting, Molecular imaging, Phosphatidylserine targeting

## Abstract

**Background:**

Tumor targeting is of high clinical and biological relevance, and major efforts have been made to develop molecular imaging technologies for visualization of the disease markers in tissue. Of particular interest is apoptosis which has a profound role within tumor development and has significant effect on cancer malignancy.

**Methods:**

Herein, we report on targeting of phosphatidylserine-exposing cells within live tumor allograft models using a synthetic near infrared zinc(II)-dipicolylamine probe. Visualization of the probe biodistribution is performed with whole body multispectral optoacoustic tomography (MSOT) system and subsequently compared to results attained by planar and tomographic fluorescence imaging systems.

**Results:**

Compared to whole body optical visualization methods, MSOT attains remarkably better imaging capacity by delivering high-resolution scans of both disease morphology and molecular function in real time. Enhanced resolution of MSOT clearly showed that the probe mainly localizes in the vessels surrounding the tumor, suggesting that its tumor selectivity is gained by targeting the phosphatidylserine exposed on the surface of tumor vessels.

**Conclusions:**

The current study demonstrates the high potential of MSOT to broadly impact the fields of tumor diagnostics and preclinical drug development.

## Background

A versatile pool of optical reporter agents and imaging methods for enhancement and probing of anatomical features and molecular function of tumors has been recently developed [1,2]. The contrast carriers reach from fluorochromes [3] and other organic dyes to quantum dots [4] and several forms of nanoparticles [5,6] with a large selection of accumulation, targeting and activation mechanisms [7,8]. It has been long realized that apoptosis plays a profound role within tumor development and has significant effect on cancer malignancy [9]. Therefore, many anticancer treatment strategies are based on the induction of apoptosis in tumors [10], and early monitoring of such treatment success is of great interest within preclinical drug development. Even though optical molecular markers of apoptosis are generally available [11,12], their *in vivo *visualization is challenging due to intensive light scattering in living tissues. Some earlier attempts to specifically image apoptotic responses in whole living animals using fluorescence molecular tomography attained generally low spatial resolution and imaging speed [13,14]. As a result, questions related to the exact probe localization (e.g., necrotic foci within the tumors versus surface of the tumor blood vessels) cannot be answered using those methods.

More recently, multispectral optoacoustic tomography (MSOT) has been emerging as a promising tool for high-resolution volumetric imaging of optical contrast in tissues [15], capable of visualizing tissue chromophores with ultrasonic resolution independent from light scattering [16]. The use of multispectral methods allows to efficiently resolve extrinsic optical agents, such as fluorochromes and fluorescent proteins [17,18]. Optoacoustic imaging correspondingly offers important advantages in small animal imaging, including the use of non-ionizing radiation, the versatile sensing of chromophoric molecules for probing cellular and sub-cellular function, good spatial resolution and, in analogy to ultrasound imaging, real-time operation [19-21].

Recently, a novel optical probe, comprised of a synthetic zinc(II)-dipicolylamine complex appended to a near-IR (NIR) carbocyanine fluorophore (subsequently called PSS-794), has been shown to selectively target anionic membrane-bound phosphatidylserine (PS) exposed by dead and dying cells within xenograft tumors in rat and mouse models [22]. Usually restricted to the inner leaflet of the plasma membrane, phosphatidylserine is selectively exposed as an early event during cell apoptosis, which is a crucial mechanism of cell number control in various physiological and pathological events [23]. Herein, we investigate whether PSS-794 can be used to image tumors *in vivo *with high resolution using MSOT.

## Methods

### Multispectral optoacoustic tomography system

**Figure 1:**
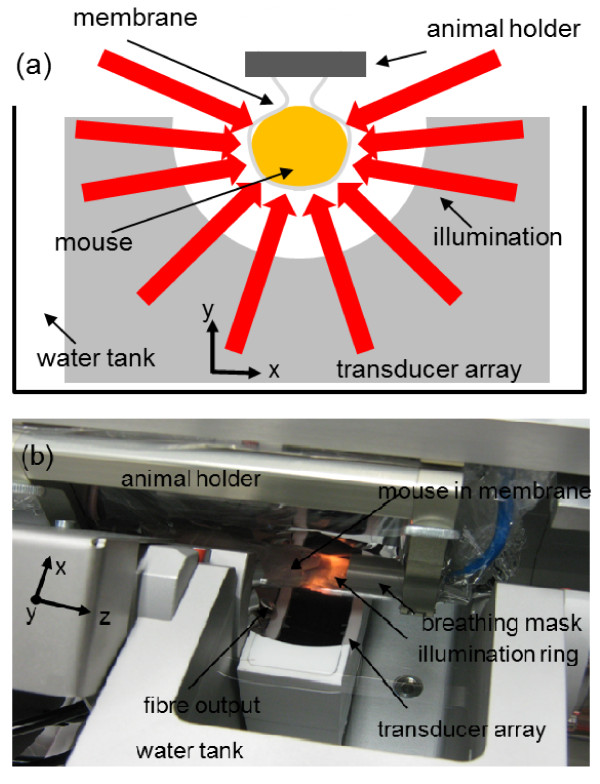
**MSOT system**. (**a**) Schematic representation of the MSOT system. A curved array of wideband and cylindrically focused ultrasound transducers enable parallel data acquisition. Optical fibers are used to homogeneously illuminate the object. A special animal holder with a transparent plastic membrane is used for animal positioning and protects the animal from direct contact with water. (**b**) Picture of a mouse during a scan in the MSOT system showing key components of the system.

A real-time MSOT scanner, similar to the one described by Razansky et al. [21], was utilized in this study (Figure 1). Briefly, it is based on an excitation source from a tuneable (680-950 nm) optical parametric oscillator laser (Phocus, Opotek Inc., Carlsbad, CA, USA) delivering < 10-ns duration pulses with repetition frequency of 10 Hz. The laser beam is guided into a silica fused-end fiber bundle (PowerLightGuide, CeramOptec GmbH, Bonn, Germany) consisting of 630 fibers partitioned into ten arms, thus, creating a ring-shaped illumination pattern of approximately 8-mm width upon the surface of the animal. The beam was sufficiently broadened to keep the laser pulse fluence on the surface of imaged objects under 20 mJ/cm^2 ^in order to meet the laser safety standards. Signal collection is based on a custom-made 64-element focused transducer array (Imasonic SaS, Voray, France) covering a solid angle of 172° around the imaged object, while the detection plane coincided with the center of the illumination ring. The individual detection elements are manufactured using piezocomposite technology with central frequency of 5 MHz, bandwidth of more than 50%, and sensitivity of approximately 18 μV/Pa and are shaped to create a cylindrical focus at 40 mm. According to ultrasonic diffraction limit and focal width at the central frequency, the effective spatial resolution of the system is estimated at 150 μm (in-plane) and 800 μm (elevational). The detected signals are digitized at 60 megasamples per second frequency by eight multichannel analog to digital converters (PXI5105, National Instruments, Austin, TX, USA) with noise floor of approximately $3.8\text{ nV}/\sqrt{\text{Hz}}$. To facilitate *in vivo *measurements, the animals were wrapped by a water-impenetrable transparent membrane that averts animal contact with water inside the imaging chamber. A linear stage (NRT150, Thorlabs GmbH, Karlsfeld, Germany) allows linear translation of the animal holder in the axial *z *direction for the acquisition of three-dimensional data sets.

### Animal imaging

All procedures involving animals and their care were conducted in full agreement with the institutional guidelines, complying with national and international laws and regulations. For imaging, four female 8-week old athymic CD-1 nude mice containing 4T1 tumor allografts were used. The tumors were obtained by injecting 0.8 × 10^5 ^4T1 cells subcutaneously into the mouse's neck where they were allowed to grow for 7 days, reaching a diameter of 0.4-0.5 cm. The PSS-794 imaging probe was prepared as previously reported [24]. The first two tumor-carrying mice were injected intravenously via the tail vein 24 h prior to imaging with 6 mg/kg (100 nmoles) and 3 mg/kg (50 nmol) of the probe, respectively. A third mouse was injected 3 h prior imaging with probe amount of 3 mg/kg (50 nmol). For control, a fourth mouse was injected with 3 mg/kg (50 nmol) of indocyanine green (ICG), a non-targeted dye having spectral characteristics similar to PSS-794 in the near-infrared, 3 h prior imaging [21]. The mice were anesthetized with a mixture of ketamine and xylazine, and were placed in supine position in the animal holder. Cross-sectional multispectral optoacoustic image datasets were acquired through the tumor at six different wavelengths in the NIR window (700, 740, 760, 780, 800, and 900 nm). For quantitative readings, the inter-wavelength laser energy variations were corrected for by normalizing the data by power meter readings (FieldMaxII-TOP, Coherent GmbH, Dieburg, Germany).

### Image reconstruction and analysis

Reconstruction of single-wavelength optoacoustic images was done with interpolated matrix model inversion method [25]. Prior to inversion, the raw optoacoustic signals were bandpass-filtered between 50 kHz and 7 MHz, and deconvolved with the combined electrical impulse response of the ultrasonic detectors and the acquisition system [26]. For inversion of the forward matrix, the iterative PLSQR algorithm has been used [27]. To selectively detect the biodistribution of the probe over intrinsic tissue absorption background, multispectral un-mixing by independent component analysis (ICA) [28] was applied.

This technique is based on the assumption that the source components of the mixed multispectral dataset are statistically independent. In order to identify these source components, the algorithm seeks a transformation of the dependant multispectral dataset into a set of independent variables which is accomplished by maximizing their non-Gaussianity because, according to the central limit-theorem, statistical independent variables are less Gaussian than their mixed dependent counterparts. The measured absorption spectra of the probe and oxygenized and deoxygenized blood were fed to the algorithm as a starting point for a guided un-mixing. The probe component was identified by comparing the resulting spectra from the un-mixing algorithm with the measured probe spectra. In contrast to other methods based on least-square un-mixing, which try to fit an *a priori *known spectra to the data, the ICA algorithm has more degrees of freedom which reduces the cross talk and leads to generally better performance.

### Cross-validation with fluorescence measurements

For validation purposes, one mouse was also imaged *ex vivo *with a newly developed 360° free-space FMT-XCT system which combines fluorescence molecular tomography (FMT) with small animal X-ray CT (XCT) into a hybrid imaging device [29]. The system was capable of simultaneous three-dimensional visualization of small animal anatomy and biodistribution of fluorescent probes *in vivo*. For volumetric FMT reconstructions, 12 angular projections distributed over full 360° range were acquired close to the excitation and emission wavelengths of PSS-794. Following the FMT-XCT measurement, the euthanized mice were cooled to -80°C. For further verification of the *in vivo *MSOT and FMT images, cross-sectional color photographs (RGB) and fluorescence images were also obtained using a cryotome supplemented by a home-built multispectral epi-fluorescence system [30]. The system consisted of a white light source and a sensitive CCD camera with motorized filter wheels for selection of the excitation (740 nm, 40 nm bandpass filter) and emission (785 nm longpass filter) wavelengths.

### Histology

After cryo-slicing, selective tissue samples in the tumor area were used for histological validation for highly specific detection of apoptosis using commercially available annexin V antibody (Abcam PLC, Cambridge, United Kingdom). The appearance of PS residues (normally hidden within the plasma membrane) on the surface of the cell is an early parameter of apoptosis, which can also be used to detect and measure apoptosis. During apoptosis, PS is translocated from the cytoplasmic face of the plasma membrane to the cell surface. Annexin V has a strong Ca2+-dependent affinity for PS and, therefore, can be used as a probe for detecting apoptosis.

## Results

**Figure 2:**
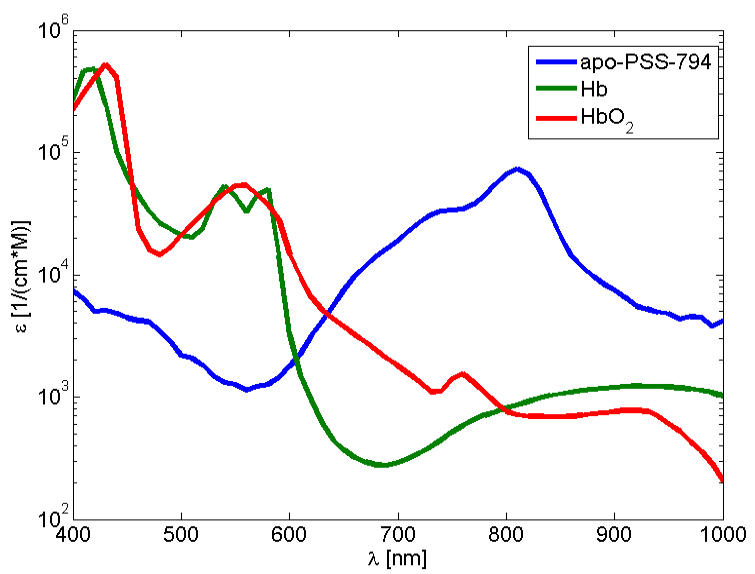
**Molar extinction coefficient of PSS-794**. In 35 mg/ml albumin along with extinction spectra of oxygenized and deoxygenized hemoglobin.

Figure 2 depicts the molar extinction coefficient of the targeted PSS-794 probe in 35 mg/ml albumin acquired using a fiber optic spectrophotometer (USB2000, Ocean Optics Inc., Dunedin, FL, USA) as well as the spectra of oxygenized and deoxygenized hemoglobin. The spectrum of PSS-794 has a characteristic shape with an absorption maximum at 810 nm close to the isosbestic point of blood. It, thus, differs significantly from the major background tissue absorbers, making it well suited for multispectral optoacoustic detection with high sensitivity.

**Figure 3:**
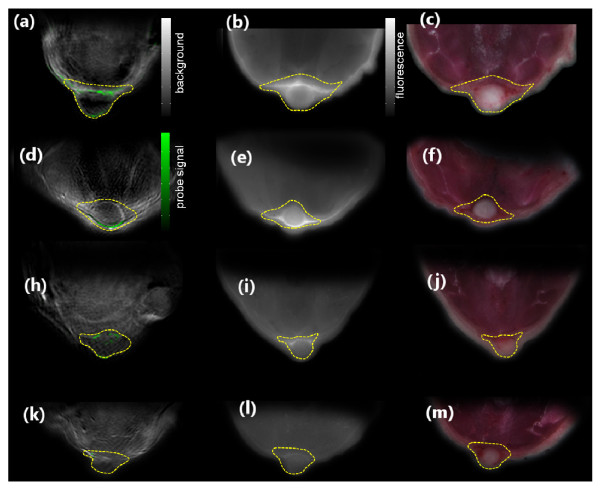
**Imaging of targeted apoptotic marker (PSS-794) in 4 T1 tumor-bearing mice**. In the first column, the MSOT images are shown. Images (**a**) and (**d**) show superposition of a single-wavelength (anatomical) optoacoustic image (in gray scale) and the unmixed component corresponding to the PSS-794 signal in color for the two mice imaged 24 h after injection. (**h**) shows the unmixed PSS-794 signal for the mouse imaged 3 h post injection, and (**k**) the ICG signal also imaged 3 h post injection. The second column shows the corresponding epi-fluorescence images of cryo-slices through the tumor. Color photograph of cryo-slices are shown in the third column. The tumor area is delineated in yellow.

Figure 3a, d show the spectrally unmixed MSOT signals from the two mice imaged *in vivo *24 h after injections. The single wavelength optoacoustic images acquired at 900 nm are shown in gray scale, while the spectrally unmixed MSOT signals are superimposed in color. A transparency threshold of 65% was applied to the unmixed data when superimposed to the single wavelength background image. The boundary of the tumor is delineated in yellow. Figure 3h shows the result from the mouse imaged 3 h after injection, and Figure 3k the result for the mouse injected with ICG also imaged 3 h after injection. The PSS-794 signal is visible in all the three cases, whereas signal from ICG is not detectable. Apparently, analysis of the high-resolution MSOT images clearly shows that the PSS-794 probe mainly accumulates in the blood vessels surrounding the tumors, while no infiltration of the probe into the tumor mass occurs.

**Figure 4:**
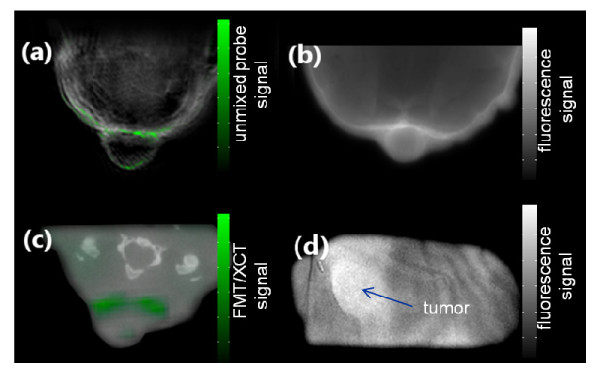
**Comparison between optical imaging and MSOT**. Image (**a**) shows the MSOT image, and (**b**) the corresponding epi-fluoresence image. An FMT-XCT reconstruction is shown in (**c**) and a planar transillumination image in (**d**)

**Figure 5:**
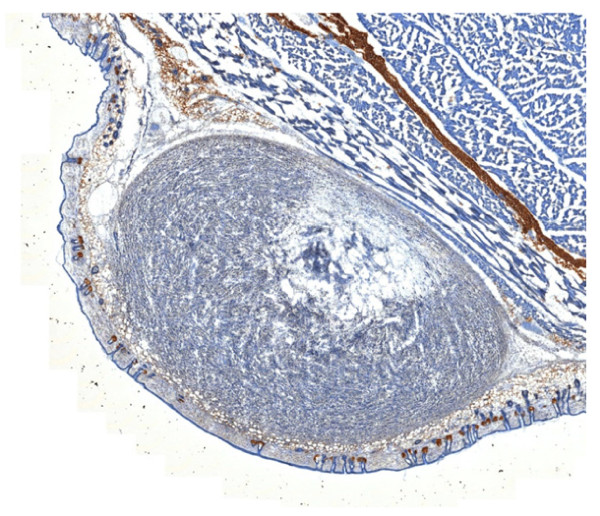
**Representative histological slice of the tumor mass**.

On the other hand, reconstruction of the subsequent *ex vivo *FMT measurement, shown in Figure 4c, generally confirms the fact that the probe specifically accumulated around the tumor areas. Yet, due to the lower resolution of the method, it is difficult to determine whether the fluorescence signal is originating only from the vasculature or from within the tumor parenchyma. The exact origin of the fluorescence signal is also not recognizable from the poorly resolved transillumination fluorescence image (Figure 4d). The results of the validation cryo-slicing and epi-fluorescence studies, made on euthanized mice, are shown in Figure 3b, e, i, l. The color pictures of the cryo-sliced mouse in Figure 3c, f, j, g shows the actual location of the tumor mass, which can also be readily delineated on the single-wavelength optoacoustic images. Observation of the brown spots in the histological image in Figure 5 suggests that only a relatively small amount of apoptotic tissue exists in the tumor.

In summary, both the *in vivo *MSOT and the *ex vivo *epi-fluorescence images clearly reveal that the fluorescence signal of the PSS-794 probe is localized in the blood vessels surrounding the tumor. This observation agrees well with literature which shows that, once there is only a small amount of apoptotic tissue in this tumor cell line, PS-targeting probes will be only observed in the tumor blood vessels [31].

## Discussion

Previous studies have shown that the fluorescent PS-targeting probe, PSS-794, can be used to optically image apoptotic/necrotic tissues often found inside tumors [22]. Here, it is demonstrated for the first time that PSS-794 also allows imaging of 4T1 tumor allografts without an actual infiltration of the probe into the tumor mass. In our experiments, lack of depth resolution of planar fluorescence imaging might have mistakenly led to the conclusion that PSS-794 is concentrated in the tumor mass. Even the use of three-dimensional optical tomography (FMT) has not attained the sufficient spatial resolution that would determine the precise location of the probe. In contrast, the much higher resolution of the MSOT revealed accumulation of the PSS-794 in the blood vessels surrounding the tumor area and clearly showed no infiltration into the tumor mass. This lack of extravasation has been also confirmed by the epi-fluorescence images made on the cryo-sliced mouse. This suggests that the vasculature of the tumor was not leaky.

The lack of extravasation is not surprising in this case since both probes are known to associate with serum proteins, and it is hard for the large protein/dye complex to leak from the neovasculature, especially if there is a high interstitial pressure in the tumor [32]. On the other hand, since the unspecific ICG dye is not detected in the tumor area while PSS-794 stays in the tumor vessels much longer, we conclude that the PSS-794 is still targeting a certain biomarker most likely the PS exposed on vessel walls of the neovasculature [33,34]. There is evidence that 20-40% of the blood vessel surfaces in all tumors, and metastases that are larger than 1 mm expose PS even though these endothelial cells are vital and not apoptotic [35]. Moreover, it normally takes 6-24 h to exert the enhanced permeability and retention (EPR) effect [36]. Thus, the strong retainment of PSS-794 at the tumor by 3 h (Figure 3h) cannot be explained either by the EPR. This is because, if the tumors were leaky, both PSS-794 and ICG should have been observed within the tumor parenchyma.

The PS-binding antibody Bavituximab^® ^(a human chimeric version of the murine monoclonal IgG_3 _antibody, 3G4) is especially effective at targeting this class of exposed PS, and it induces impressive antitumor activity in a wide array of tumors with no evidence of toxicity [37,38]. This raises the idea that zinc(II)-dipicolylmine complexes may have value as near-universal targeting agents for imaging and treatment of tumors; however, more studies are needed to test this interesting hypothesis. The current study showcases the power of MSOT to uncover new high resolution information about the precise location of imaging probes within sites of disease.

## Conclusions

In conclusion, we used MSOT to accurately detect the targeted PSS-794 probe *in vivo *over strong background absorption of blood with spatial resolution on the order of 150 μm, attaining unprecedented image quality for deep-tissue imaging of optical contrast. The enhanced resolution of the MSOT clearly showed that the probe mainly localizes in the vessels surrounding the tumor, suggesting that the probe gains its tumor selectivity by targeting the PS that is exposed on the surface of the tumor blood vessels. The MSOT results further correlated well with cryo-slices and epi-fluorescence images of *ex vivo *specimens. Overall, this study demonstrates the high potential of MSOT to broadly impact the fields of tumor diagnostics and preclinical drug development.

## Competing interests

The authors declare that they have no competing interests.

## Authors' contributions

AB, EH, VN and DR designed the experiments. AB and EH performed the data analysis. AA did the FMT-XCT imaging. AB, DR and BDS have written the manuscript. BDS provided the PSS-794 contrast agent. DR and VN provided conceptual input and supervised the research. All authors read and approved the final manuscript.
